# Complicated Posterior Cervical Skin and Soft Tissue Infections at a Single Referral Center

**DOI:** 10.1155/2020/5230763

**Published:** 2020-08-04

**Authors:** Jing-Chun Zhao, Xiu-Hang Zhang, Nan Zhang, Zhen-Dong Wu, Jian Wang, Qing-Hua Yu, Lei Hong, Kai Shi, Jia-Ao Yu

**Affiliations:** Department of Burn Surgery, The First Hospital of Jilin University, Changchun 130021, China

## Abstract

**Background:**

Although the incidence and mortality of complicated skin and soft tissue infections have decreased, this infection is still relatively frequent and can be associated with lethal complications. In this study, the authors present our clinical experience of patients with complicated posterior cervical skin and soft tissue infections (CPCSSTIs) diagnosed and treated in a reconstructive unit in northeastern China.

**Methods:**

A retrospective chart review of patients diagnosed with CPCSSTIs from January 2009 to December 2018 was performed. To make the results objective and convincing, a data analysis was performed relating to demographic characteristics, clinical presentation, predisposing factor, bacterial culture, laboratory and radiographic evaluations, diagnostic clues, management, and complications as well as the clinical course and outcome.

**Results:**

During the ten-year period, there were 174 consecutive patients admitted to our reconstructive center with final diagnosis of CPCSSTIs included. All the patients were adults, and the majority were male (67.2%). The patient's mean age was 51.3 years (range, 15-88 years). There were 114 patients (65.5%) that had associated systemic diseases, with diabetes mellitus (40.2%) as the most common predisposing factor. Common presented clinical symptoms were pain (90.8%), swelling (85.1%), and erythema (77%) of the neck. Surgical treatment was performed in all the patients, and most of them (83.9%) received the first surgery within 24 h. The most commonly isolated pathogen was *Staphylococcus aureus* (30%). Vancomycin (21.3%) was the most commonly used antibiotics, followed by cefepime (18.4%). All the patients survived and were discharged with a mean duration of hospitalization of 28.7 days. Those patients with predisposing factors (31.4 ± 12.35 days) or complications (41.0 ± 12.5 days) tended to have a longer hospital stay. The mean total costs of admission per patient were 47 644 RMB.

**Conclusion:**

This study highlights the high cost burden of CPCSSTI patients. Those patients with predisposing factors or complications tended to have a longer hospital stay.

## 1. Introduction

Skin and soft tissue infections (SSTIs) are an important cause of morbidity and mortality among hospitalized patients and a major therapeutic challenge for clinicians that include a variety of pathological conditions that involve the skin and underlying subcutaneous tissue, fascia, or muscle [1]. Although the mortality rate of SSTIs has decreased significantly compared with that of early reports since the improvement of modern diagnostic techniques, widespread availability of antibiotics, critical patient support modalities, and early surgical intervention, however, SSTIs remain to cause significant morbidity and mortality rates and present an important challenging problem [2].

Various classification systems have been used to describe SSTIs including variables such as anatomic location, causative pathogens, rate of progression, depth of infection, and severity of clinical presentation [1]. Generally, SSTIs can be classified as uncomplicated and complicated infections. Uncomplicated SSTIs include superficial infections of the skin, subcutaneous tissues, and superficial fascia, which required antibiotics or surgical incision for drainage of abscess alone. In contrast, complicated SSTIs (CSSTIs) include the deeper neck tissues, which extend below the dermis and may involve the subcutaneous tissue, fascial planes, or muscular compartments presenting as complex abscesses (carbuncle), necrotizing fasciitis, or myonecrosis and subsequently require surgical intervention for drainage and/or debridement [1, 3, 4].

The complicated posterior cervical skin and soft tissue infections (CPCSSTIs) are fairly common in clinical settings and are a source of considerable hospitalization, substantial health care costs, and mortality [5–7]. Many etiological or predisposing factors are very important in the prevalence of CSSTIs and modification of its outcome, which may result in death. Typically, CSSTIs develop in patients with diabetes mellitus (DM), immunosuppression, cirrhosis, cardiovascular disease, elderly age, history of radiation and/or chemotherapy, chronic renal failure, HIV, cancer, peripheral vascular disease, intravenous drug abuse, or alcoholism [8, 9]. In addition, timing of CSSTIs onset to admission may play a prognostic role in severe cases which may stratify patients with similar disease severity into different mortality risk groups. Therefore, with increasing frequency of antibiotic resistance, relative frequency, and their serious complications, CPCSSTIs should not be ignored, particularly in immunocompromised patients and those with low socioeconomic status [10, 11].

Prompt recognition, timely and accurate diagnosis with laboratory and imaging tools, adequate and appropriate antimicrobial therapy, early radical surgical drainage or debridement, and management of complications are the keys to a successful treatment for CSSTIs.

Previous retrospective analyses have been done on patients with CSSTIs, concentrating on the infection located in the potential spaces or fascial planes of the anterior neck. No recent information on the situation in the incidence, severity, causes, and occurrence of complications in CPCSSTIs is available which may have their special characteristic. The main purpose of the present study was to present our own experience of the clinical course of CPCSSTIs to identify clinical features and emphasize the importance of appropriate treatment selection in those patients.

## 2. Methods

### 2.1. Trial Design

This was a retrospective study performed with strict discretion in the Department of Burns, The First Hospital of Jilin University, Changchun, China. Ethical approval to conduct the study was obtained from the Institutional Review Board from The First Hospital of Jilin University. All data was anonymized without any personally identifiable information.

### 2.2. Participants

All available medical records of patients treated for SSTIs at the authors' institution (a tertiary referral academic center) in northeast China, during a 10-year period (between January 2009 and December 2018), were retrospectively investigated. Patients of all age groups and both genders were included. Only patients with CPCSSTIs (major posterior cervical abscesses or carbuncle, infections deeper than the layers of deep cervical fascia or potential spaces between them) were included in this study. The diagnosis of CPCSSTIs was based on clinical presentations and/or imaging studies (computed tomography (CT), X-ray, or neck sonography), laboratory results, or surgical findings. Patients with anterior neck infections, uncomplicated posterior cervical SSTIs, infections due to external neck injuries (traumatic or surgical), associated with tuberculosis, and infections in head and neck malignant tumors were excluded. Those who did not complete the treatment or cases with incomplete data were also excluded.

### 2.3. Management of CPCSSTIs

In our department, the treatment protocol had been formulated as follows: when a patient with the suspicion of CPCSSTIs was admitted, we performed thorough physical examination and peripheral blood laboratory tests. Imaging studies such as neck soft tissue radiography or neck CT scan were used selectively. A paired swab and tissue culture had been obtained for the identification of underlying microorganisms. Initial empirical intravenous antibiotics were immediately administered before the culture results were available. Thereafter, the treatment regimen and dosage of antibiotic agents were later modified based on the bacterial culture and sensitivity report.

When the diagnosis was established or highly suspected, prompt surgical debridement followed by hospital admission was performed. Wound dressings were changed every day following the surgery, or negative pressure wound therapy (NPWT) was applied after the infected and necrotic tissue of the wound was completely removed. NPWT was changed every three to five days until closure of the wound was performed in the second stage. For patients with signs and symptoms of bacteremia, sepsis, or septic shock at presentation, critical patient care in intensive care unit was administered and surgery was postponed. Hypoalbuminemia or anemia was corrected with intravenous injection of 20% albumin solution or blood transfusion to maintain serum albumin greater than 30 g/L or hemoglobin greater than 100 g/L.

### 2.4. Measured Variables and Data Collection

Data of the following clinical variables were reviewed and grouped into (a) demographic information: age, sex, seasonal distribution, date, and year of presentation; (b) predisposing factors; (c) clinical features: length of the disease from the first symptoms to the date of admission, history of previous antibiotic use before hospitalization, clinical manifestation and physical examination signs, and subcategory of infection (carbuncle or necrotizing fasciitis); (d) diagnostic procedures: clinical, laboratory data, bacteriology, and imaging studies; (e) treatment protocol, complication, and outcome: antibiotic use during hospitalization, type and number of surgical procedures, length of hospitalization, and economic burden of the patients, complications, and overall survival.

The presence of predisposing factor of each patient, including the patients' medical history of hypertension, DM, chronic liver disease, chronic pulmonary disease, end-stage/chronic renal disease, and coronary artery disease, was reviewed.

Clinical symptoms and signs of CPCSSTIs, including fever, neck pain, restricted movement of neck, neck swelling and neck skin fistulization, neck erythema, and localized increase in temperature, were collected.

The laboratory data were obtained from all the patients on the day of admission including peripheral blood white blood cell (WBC) count, neutrophil count, hemoglobin, albumin, blood glucose, and serum C-reactive protein (CRP). The long-term control state of DM was evaluated using the glycated hemoglobin (HbA1c) level (HbA1c:<7.0%, good control; >7.0%, poor control). Blood culture was performed in cases with repeated fever with body temperature greater than 38.0°C or with signs of bacteremia, sepsis, or septic shock. Purulent samples were taken and examined in surgical patients from swabs of the wound and sent for aerobic wound cultures and antibiotic sensitivity with strict asepsis.

Imaging examination, including neck soft tissue radiography or CT scan, was used to identify the anatomical extension, characteristics of the infection (purulent or not purulent), and depth of infection when necrotizing fasciitis was highly suspected, extensive deep intermuscular fascia was involved, no response to initial treatment, or guided surgical drainage procedures.

Two subcategories of deep neck infections were established according to their character as previously reported [6]. Accordingly, patients with an evident abscess pockets in the posterior deep cervical space were defined as the “deep cervical abscess” group, whereas those suffering from severe necrosis with soft tissue infection spreading along the posterior deep cervical fascia and cervical muscles were defined as the “necrotizing fasciitis” group. Cervical necrotizing fasciitis was confirmed by histologic examination and operative report documentation.

Complications of CPCSSTIs identified in these patients during the clinical course were defined as sepsis, septic shock, or bacteremia that are all serious complications that justify the importance of this pathology requiring intensive care in addition to antibiotic administration and/or initial surgical intervention during the initial hospitalization.

The overall results of this study were expressed as numbers and percentages of cases for categorical variables, means ± standard deviation (SD), and ranges of values for continuous variables. The findings were compared to those in the available literature.

## 3. Results

### 3.1. Patient Demographics

**Figure 1:**
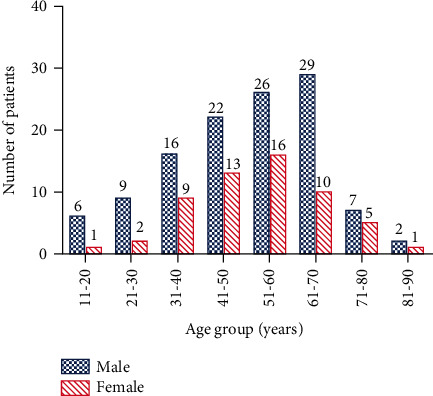
Age and sex distribution of patients.

**Figure 2:**
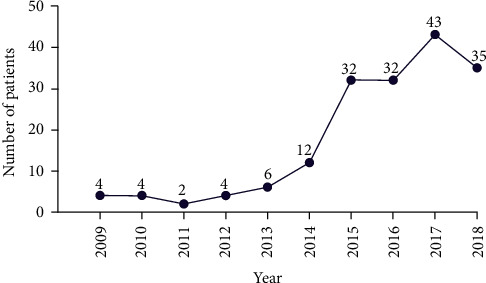
Annual distribution of posterior cervical complicated skin and soft tissue infections.

**Figure 3:**
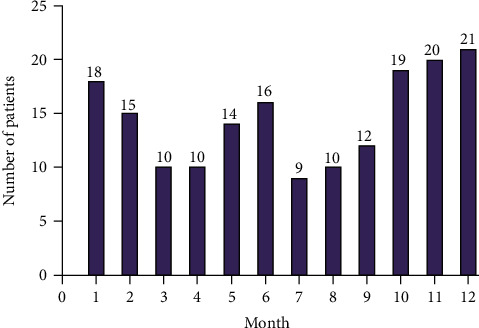
Distribution of the patients throughout the years (month distribution).

During the 10-year study period, a total of 210 patients with posterior cervical SSTIs were identified, and thirty-six cases were excluded from the study (nine patients were diagnosed with cellulitis, five patients were suffer from furuncles, incomplete data were found in 10 patients, five patients did not complete the treatment, and cervical **infections due to trauma were noted in seven patients**). Ultimately, a cohort of 174 eligible cases was enrolled into this study. Of them, 117 were males (67.2%) and 57 were females (32.8%), with a male-to-female ratio of 2.05/1. The mean age of the patients was 51.3 ± 15.6 years (range, 15–88 years), with the age distribution of patient curve peaked in the fifth decade of life followed by sixth, 42 (24.1%) and 39 (22.4%), respectively. The age and sex distribution are shown in Figure 1. Among the 174 patients, 91 (35.6%) were working persons, and 83 (48.9%) were nonworking (students, unemployed persons, and retired people). Throughout the study period, the number of inpatients treated was steady during the first four years; however, it showed an upward trend in the number of presented cases since 2013; while there were four cases of CPCSSTIs in 2009, it increased to 35 cases in 2018, with peak prevalence in 2017 (43 cases). The chronological distribution of the CPCSSTI patients is showed in Figure 2. The number of CPCSSTI inpatients was highest in December (21 patients) and lowest in July (9 patients) (Figure 3). The most common season for CPCSSTIs was winter with 54 patients involved (31.03%), followed by fall (28.74%), summer (20.69%), and spring (19.54%), with 50, 36, and 34 patients involved, respectively. CPCSSTI was found year-round; however, winter seemed much more common.  Table 1 shows the baseline demographics of all the patients.

### 3.2. Predisposing Factor

There were 114 patients (65.5%) who had predisposing risk factors; 81 were men, and 33 were women, with a mean age of 52.9 ± 14.35 years. In this patient group, the most common predisposing factor was type-2 DM in 70 patients (40.2%), followed by hypoalbuminemia in 62 patients (35.6%) and anemia in 56 patients (Table 2). Ninety-five patients (54.6%) were smokers, and 58 patients (33.3%) were alcoholics. There was no case of known intravenous drug abuse or known primary or acquired immunodeficiency.

### 3.3. Clinical Features

**Figure 4:**
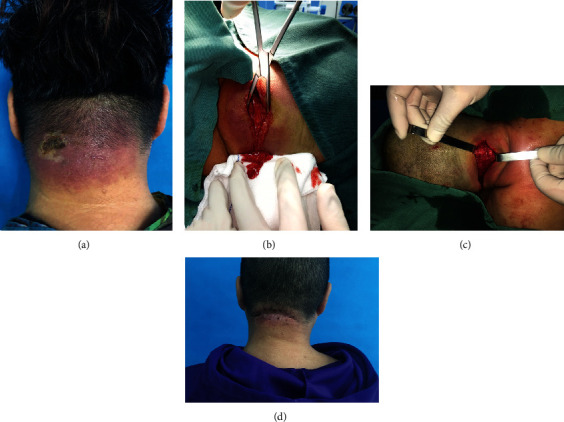
A 58-year-old female with a history of diabetes mellitus, who was afebrile with hard, erythematous, and hot swelling in the posterior region of the neck with spontaneous drainage of purulent fluid without showing crepitus. (a) Picture of wound on admission. (b) Incision and drainage was performed immediately. (c) Surgical wound after complete debridement. (d) Final outcome after suture of the wound. The incision healed well.

**Figure 5:**
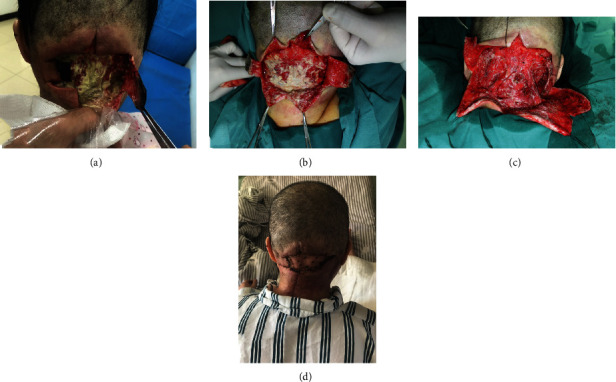
A 50-year-old female with a history of diabetes mellitus, who complained of swelling and pain in the posterior cervical area. Inadequate incision was performed in regional hospital and visited our institution for definitive treatment. Diagnosis of necrotizing fasciitis was confirmed by the findings during the surgery and histological report. (a) Wound at presentation. (b) Intraoperative picture of the patient. Aggressive debridement was performed immediately. (c) Necrotic tissue remained existent following the initial debridement, and second debridement was performed. (d) The incision healed completely, and the skin graft took well.

Posterior neck abscess (carbuncle) formation was noted in 170 patients (97.7%) and cervical necrotizing fasciitis in only four patients (2.3%). Each one of the representative cases was shown in Figures 4 and 5.

The mean time from symptom onset to the demand for health services was 11.0 ± 6.9 days (range, 1-30 days). The most common presenting symptoms on admission were neck pain (158 cases, 90.8%), followed by local neck swelling and had a progressive increase in size in the last few days, and neck erythema, documented in 148 (85.1%) and 134 (77%) patients, respectively. The mean body temperature at presentation was 37.2 ± 0.79°C (range, 36.5-39.6°C). A summary of the clinical features of the patient cohort are presented in Table 3.

## 4. Diagnostic Procedure

### 4.1. Laboratory Findings

Regarding the laboratory test findings, the mean ± SD values at the time of admission were WBC count = 13.18 ± 8.08 × 10^9^/L (range 1.87–39.95 × 10^9^/L) and neutrophil count = 10.88 ± 7.71 × 10^9^/L (range 1.14–29.29 × 10^9^/L) in all the 174 cases with available data, respectively. In addition, leukocytosis (WBC > 12.0 × 10^9^/L) was detected in 46 patients (26.1%) and leukopenia (WBC < 4.0 × 10^9^/L) in six patients (3.4%). The results of systemic inflammatory response syndrome (SIRS) and quick sequential organ failure assessment (qSOFA) score were calculated and summarized in Tables 4 and 5. Twenty-four patients (13.8%) had a WBC count of more than 20.0 × 10^9^/L. Hemoglobin = 119.3 ± 23.66 g/L (range, 50–179 g/L) in all the 174 cases, with a lower than 120 g/L in male or 110 g/L in female, was found in 56 patients (32.2%). Serum albumin = 29.70 ± 7.12 g/L (range, 12.7–44.2 g/L) in 166 of 174 cases, with hypoalbuminemia (<30 g/L), was found in 62 patients (37.3%). Blood glucose = 9.79 ± 5.61 mmol/L (range, 3.66–28.38mmol/L) in 170 of 174 cases, with an elevation of blood glucose (>11.1 mmol/L), was found in 40 patients (23.5%). Glycated hemoglobin = 10.06 ± 2.72% (range, 5.2–14.5%) in 76 of 174 patients, with poor control of blood glucose, was found in 33 patients (>7%). CRP = 155.89 ± 116.10 mg/L (range, 2.7-525.0 mg/L) in 146 of 174 cases, with 68 cases (46.6%), had the elevation of the CRP level (>3.5 mg/L).

Blood cultures were routinely checked for patients with signs of sepsis (16 cases), resulting positive cultures in 7 patients, with the most prevalent occurrence of *Klebsiella pneumoniae* (2 cases). Laboratory data at admission is presented in Table 6.

### 4.2. Bacteriology

The results of bacterial cultures were available for 150 of the 174 cases who underwent surgical treatment or wound swabs (86.2%). Bacteriological samples were missing in 24 patients. There were 111 of those patients (74%) who had identifiable bacterial growths, and the remaining 39 cultures showed no bacterial growth (26%). The most commonly isolated organisms of the positive culture were *Staphylococcus aureus* (*n* = 45, 30%; including 4 methicillin-resistant *S. aureus* (MRSA)), followed by *Escherichia coli* (*n* = 15, 10%) and *Klebsiella pneumoniae* (*n* = 11, 7.3%). Polymicrobial culture was detected in 13 patients (8.7%, 2–3 species). The microbiology findings are summarized in Table 7.

### 4.3. Imaging Findings

In the current study, only a few patients underwent imaging: 21 cases (12.1%) had ultrasound scanning, 4 cases (2.3%) underwent neck soft tissue X-ray, and 6 patients (3.4%) underwent CT scan. None of the patients underwent magnetic resonance imaging (MRI) or other imaging examination.

### 4.4. Treatment Protocol, Complication, and Outcome

Thirty-four out of 174 patients (76.5%) had been previously treated before admission, 16 patients (9.2%) underwent surgical intervention combined with antibiotic therapy in regional hospital, and 18 patients (10.3%) were already treated with antibiotics alone, orally or intravenously, in regional hospital, clinics, or by themselves. Notably, thirteen patients (7.5%) used traditional Chinese medicine topically by themselves prior to their first visit to our institution.

After admission, all the patients initially received broad-spectrum intravenous antimicrobial therapy empirically at their presentation, in order to cover the majority of aerobic, gram-negative, and gram-positive organisms involved in CPCSSTIs. The antimicrobial agents and their doses were then adjusted according to microbiological bacterial culture and clinical response.

The most common treatment regimens of empirical antibiotics were vancomycin (*n* = 38, 21.3%), followed by cefepime (*n* = 32, 18.4%) and meropenem (*n* = 31, 17.8%), cefuroxime (*n* = 26, 14.9%), ceftriaxone (*n* = 19, 10.9%), and ornidazole (*n* = 14, 8.0%). Other antibiotics used in this study group were as follows: levofloxacin (*n* = 8, 4.6%), cefoperazone tazobactam (*n* = 6, 3.4%), tigecycline (*n* = 5, 2.9%), imipenem (*n* = 4, 2.3%), mezlocillin sodium and sulbactam sodium (*n* = 4, 2.3%), teicoplanin (*n* = 3, 1.7%), moxifloxacin (*n* = 3, 1.7%), penicillin sodium (*n* = 2, 1.1%), ceftezole (*n* = 1, 0.6%), flucloxacillin sodium (*n* = 1, 0.6%), sulfoxil sodium (*n* = 1, 0.6%), cephalosporin (*n* = 1, 0.6%), and doxycycline (*n* = 1, 0.6%).

In this study, all the patients underwent aggressive surgery, including debridement of necrotic tissue, primary suture and drainage, and secondary skin grafting. Most of the patients (146/174, 83.9%) received the first surgery within 24 h. The mean time interval from hospital admission until surgery was 1 day (range, 0-5 days). Seventy-six patients (43.7%) were subjected to a series of surgeries (2-4 operations). The reason for multiple surgeries was identified in 68 patients, including poor control of DM (21 cases), age 70 years or older (16 patients), inadequate debridement (9 cases), necrotizing fasciitis (4 cases), polymicrobial infection (4 cases), MRSA (2 cases), malnutrition (6 cases), pneumonia (3 cases), chronic renal failure (2 cases), and hematological diseases (1 case). Skin grafting was performed in 62 cases when healthy granulation tissue of the debrided wound was present and wound swab culture returned negative, with scalp or thigh as the most common donor site (14 and 48 cases, respectively). No morbidity of the donor site was noted.

In addition, supportive medical treatments was also initiated when required, which included fluid therapy, analgesics, nutritional support, blood transfusion, control of blood glucose, and antipyretics. When patients with decompensated systemic diseases were involved, appropriate specialists, mostly endocrinologist, cardiologist, or internal medicine specialists, were consulted regarding the therapy.

Life-threatening complications were developed in seven cases (4.0%), including bacteremia in four cases and septic shock in three cases. All the patients were discharged in stable condition, with the mean length of stay 28.7 ± 12.95 days (range, 12-63 days). None of our patients died of CPCSSTIs or its complication. Those patients with predisposing factors (31.4 ± 12.35 days) or complications (41.0 ± 12.5 days) tended to have a longer hospital stay. The mean cost of admission per patient was 47 644 RMB (range, 5 848-303 579 RMB). Signs of cellulitis in two patients and carbuncle in one patient were found, and they were hospitalized again for management.

## 5. Discussion

To our knowledge, this 10-year study of 174 patients is the only study with the largest outcomes analysis of CPCSSTIs which adds to the literature of CSSTIs by looking at the characteristics of an Asian population in northeast China. Men with CPCSSTIs were predominant in the present study (117/174, 67.2%), which is similar to previous reports [8]. Higher incidence of this infection was found among patients aged between 50 and 59 years (24.1%), with a mean age of years 51.3 years, which is in keeping with the findings of a previous study [10]. A major increase in CPCSSTI incidence in our setting was noted in the last few years. If we consider the earlier (2009-2013) and later (2014-2018) periods separately, the number of patients suffering from CPCSSTIs increases significantly in the later period (earlier: 20 patients, later: 154 patients). The reason for these discrepancies remains unclear. The increasing prevalence of DM each year in Jilin Province (from 3.52% in 2007 [12] rise to 9.1% in 2019 [13]) may partly account for it because DM was the main predisposing factor of CPCSSTIs in the current study.

The results of pus cultures from either surgery or wound swab were available in 150 of 174 patients (86.2%); positive report of culture and polymicrobial growth was identified in 74% and 8.7%, respectively. From the cases that yielded positive bacterial culture in the current series, the most common microorganism identified was *Staphylococcus aureus* (45, 30%), with positive cultures of polymicrobial infection identified in 13 patients (8.7%). This result is consistent with a previous study [14]. When pus drainage was available, Huang et al. [15] suggest Gram stain and acid-fast staining of pus, instead of bacterial culture alone, as early as possible before initiating antimicrobial therapy, may be helpful for pathogens identified.

There were 39 cases (26%) that had negative cultures and only 7 out of 16 patients returned positive results of blood culture in this study. In addition to the fact that the specimens were sterile, the present results may be explained by the use of antibiotics before admission, or empirical antibiotics were commenced prior to taking blood samples and surgical intervention, possibly, or an improper sample collection may have affected the results of microbiological tests of this study. Actually, blood cultures are reported to be only positive in less than 40% of cases in other pathologies with sepsis which may indicate that blood cultures are not the most vital part of care and hence one should exercise clinical judgment with regard to antibiotic therapy. Last but not the least, the possibility of anaerobic infections cannot be ruled out. In light of the variable pathological organisms and the rising incidence of polymicrobial infections, broad-spectrum empirical antimicrobial therapy is advocated in treating CSSTIs [16].

Our series demonstrated a predominance of the infections in winter. The seasonal variation in this study may be attributed to the high incidence of diseases (predisposing factors for CPCSSTIs) in winter. Previous studies indicate that an increase of outdoor air pollutants corresponds to increases in diabetes and liver morbidity, and the effects are stronger in the cool season than those in the warm season; females and the elderly were generally more vulnerable to outdoor air pollution. In addition, the high prevalence of CPCSSTIs in winter may probably due to bathing is restricted because of the extreme cold weather in winter in northeast China. Meanwhile, some of the patients were farmers; they did not come to the hospital until the harvest in autumn was over.

Although CPCSSTIs were a kind of bacterial infection, fever was present in 64.4%, and 26.1% had WBC count of over 12 000 cells/mm^3^ in the present study. Neither fever nor leukocytosis is a constant finding in this study. An explanation for this situation may be the use of antibiotic and anti-inflammatory treatments prior to hospitalization, and the administration of empirical broad spectrum antibiotics before obtaining culture samples, which might have masked the presentations of CPCSSTIs without fever or leukocytosis.

Development of modern imaging tools has greatly facilitated the diagnosis of CPCSSTIs. Plain radiography, ultrasonography, CT, or MRI may show soft tissue edema or fascial thickening, fluid collections, or soft tissue air [17]. Surprisingly, only a relatively small number of patients in our selection underwent imaging. The reason for this discrepancy may be we diagnose the CPCSSTIs mainly depending on the clinical presentation, laboratory results, and intraoperative findings. Emergent surgery debridement was performed immediately after admission to the hospital, which is why only a small number of patients were scanned. However, we agree that imaging should always be carried out if there is any suspicion of the spread of infection towards other areas.

Our report has shown association of CPCSSTIs with DM in 70 cases (40.2%), which is similar as compared with the study of Raya-Cruz et al. (33%) [18]. In DM patients, hyperglycemia may impair several mechanisms of immune defense which result in predisposition to infection and complications, including impaired neutrophil function (e.g., chemotaxis, bacterial killing, phagocytosis, and impaired adherence), impaired myeloperoxidase activity and antioxidant system and micro- and macroangiopathies, and a decrease in cytokine response during leukotriene release in humoral immunity [19–21]. In return, lipopolysaccharide secretion by bacteria and infection-mediated upregulation of cytokine synthesis worsen diabetes [22]. Diabetes is a commonly recognized risk factor associated with the development of CSSTIs that may exhibit a significantly higher complication rate, longer length of hospital stay, and many more concurrently infected spaces than those without DM [23]. For those with DM, aggressive control of blood glucose concentration in plasma is necessary. Hirasawa et al. [24] recommend that in patients with DM, preventing aggravation by strictly controlling acute-phase glycaemia to 250 mg/dl is necessary.

According to our experience, patients with CSSTIs need to be treated as soon as possible surgically, and repeated surgery may require until debridement and drainage are complete and healing has commenced. In our study, all the patients underwent surgical treatment, and over 40% of patients required multiple surgical treatments. The high proportion of the surgical rate may be because our hospital was a tertiary center which received and treated patients with severe CPCSSTIs. In addition, only severe cases were enrolled in this study. Patients with severe CSSTIs were more likely to undergo surgical interventions [18].

There are several limitations to our study. First, as a retrospective study in nature in a single medical center, standardized documentation relating to the clinical patient evaluation and overall fitness status are hardly reach. Some data on physical examinations and laboratory results were missing which may exert a great influence on the clinical course and outcome. Second, we could not eliminate our institutional bias, and there was no standardization or protocol to determine which patients should undergo immediate surgery. The timing of surgical intervention may have been influenced by an individual surgeon decision and level of surgeon experience not captured herein. Third, this study spans in ten years with a heterogeneous patient population which may differ in infected defect and severity. Finally, our study can only reflect experiences from a reconstructive unit at a single medical center, which may make the experience and results of the present study not replicable by other general units. However, the characteristics of CPCSSTIs can still be deduced from our data. Further multicenter, prospective studies assessing the different characteristics of CPCSSTIs would be helpful to overcome these limitations.

## 6. Conclusions

Patients with DM are susceptible to CPCSSTIs. When dealing with CPCSSTIs in a high-risk group (older patients with DM or other predisposing factors), more attention should be paid to the prevention of complications and shorter length of hospital stay. Timely visit of patients is crucial for optimal treatment and warrants a better outcome, avoiding the complication and reducing the financial cost, which is of huge benefit for people who lived in economically underdeveloped areas, like most of the patients in our study group.

## Figures and Tables

**Table 1:** Patient demographics.

Demographic (*N* = 174)	Mean (SD) or *N* (%)
Sex (M/F)	117 (67.2)/57 (32.8)
Age (years)	51.3 (15.6)
Smoker	95 (54.6)
Chronic drinker	58 (33.3)
Days before admission	11.0 (6.9)
Antibiotics prior to admission	18 (10.3)
Traditional Chinese medicine prior to admission	13 (7.5)
Length of hospital stay (days)	28.7 (12.95)
Cost (RMB)	47 644 (2672)
Death during treatment	NA

SD: standard deviation.

**Table 2:** Summary of the predisposing factors^∗^.

Predisposing factor (*N* = 174)	*N* (%)
None	60 (34.5)
Diabetes mellitus	70 (40.2)
Hypertension	19 (10.9)
Pneumonia	9 (5.2)
Cardiovascular diseases	9 (5.2)
Liver diseases	6 (3.4)
Renal insufficiency	5 (2.9)
Hematological diseases	3 (1.7)
Anemia	56 (32.2)
Hypoalbuminemia	62 (35.6)
Autoimmune disease	2 (1.1)
Psychiatric disorders	1 (0.6)
Gout	2 (1.1)
Ulcerative colitis	1 (0.6)
Upper gastrointestinal hemorrhage	1 (0.6)
Congestive heart failure	1 (0.6)

^∗^Given patient could have more than one risk factor.

**Table 3:** Patients presenting characteristics of posterior deep neck infection^∗^.

Clinical characteristics	*N* (%)
Neck pain	158 (90.8)
Neck swelling	148 (85.1)
Neck erythema	134 (77.0)
Localized increase in temperature	128 (73.6)
Fever	112 (64.4)
Restricted neck movement	86 (49.4)
Neck skin fistulization	52 (29.9)
Inadequate incision and drainage of neck abscess	16 (9.2)

^∗^Given patient might present more than one symptom/sign.

**Table 4:** Score of patients by SIRS criteria measured at admission.

SIRS criteria	Number of SIRS criteria present	*N* (%)
(i) Temperature > 38°C or< 36°C (ii) Heart rate > 90/min (iii) Respiratory rate > 20/min or PaCO_2_ < 32 mm Hg (4.3 kPa) (iv) White blood cell count > 12, 000/mm^3^ or <4,000/mm^3^ or >10% immature bands	0	83 (47.2)
	1	42 (23.9)
	2	22 (12.5)
	3	17 (9.7)
	4	12 (6.8)

SIRS: systemic inflammatory response syndrome; PaCO2: partial pressure of carbon dioxide.

**Table 5:** Score of patients by qSOFA criteria measured at admission.

qSOFA criteria	Number of SIRS criteria present	*N* (%)
(i) Altered consciousness (ii) Respiratory rate ≥ 22/min (iii) Systolic blood pressure ≤ 100 mm Hg	0	140 (79.5)
	1	18 (10.2)
	2	11 (6.3)
	3	7 (4.0)

qSOFA: quick sequential organ failure assessment.

**Table 6:** Laboratory data at admission.

Laboratory parameters (reference range)	Mean (SD)
White blood cells count (×10^9^/L, 3.5-9.5)	13.18 (8.08)
Neutrophil count (×10^9^/L, 1.8-6.3)	10.88 (7.71)
C reactive protein (mg/L, 0-3.5)	155.89 (116.10)
Blood glucose (mmol/L, 4.1-5.9)	9.79 (5.61)
Glycated hemoglobin (%, 4.27-6.07)	10.06 (2.72)
Hemoglobin (g/L, 115-150)	119.3 (23.66)
Serum albumin (g/L, 40-55)	29.70 (7.12)
Blood culture (case)	
*Klebsiella pneumoniae*	2
*Streptococcus viridans+Escherichia coli*	1
*Enterococcus*	1
Methicillin-resistant *S. aureus*	1
*Staphylococcus aureus*	1
*Pseudomonas aeruginosa*	1
No growth	9

SD: standard deviation.

**Table 7:** Bacteriology in patients with posterior deep neck infection.

Microorganisms (*N* = 150)	*N* (%)
Gram-positive	
*Staphylococcus aureus*^∗^	45 (30)
*Streptococcus viridans*	3 (2)
*Coagulase-negative staphylococci*	4 (2.7)
*Streptococcus pyogenes*	2 (1.3)
*Enterococcus*	3 (2)
*Streptococcus anginosus*	4 (2.7)
*Staphylococcus haemolyticus*	1 (0.7)
*Micrococcus kristinae*	1 (0.7)
*Staphylococcus epidermidis*	1 (0.7)
*Streptococcus constellatus*	1 (0.7)
*Streptococcus agalactiae*	1 (0.7)
Gram-negative	
*Escherichia coli*	15 (10)
*Klebsiella pneumoniae*	11 (7.3)
*Pseudomonas aeruginosa*	3 (2)
*Enterobacter cloacae*	9 (6)
*Corynebacterium*	1 (0.7)
*Actinobacillus*	1 (0.7)
*Acinetobacter baumannii*	2 (1.3)
*Proteus mirabilis*	1 (0.7)
*Stenotrophomonas maltophilia*	1 (0.7)
*Enterobacter aerogenes*	1 (0.7)
Mixed flora^#^	13 (8.7)
No growth	39 (26)
Total	150 (100)

^∗^Includes 4 cases of methicillin-resistant *S. aureus. ^#^*Includes *Streptococcus anginosus*, *Escherichia coli*, *Pseudomonas aeruginosa*, *coagulase-negative Staphylococci*, *Streptococcus viridans*, *Staphylococcus aureus*, *Enterococcus*, *Klebsiella pneumoniae*, *Enterobacter cloacae*, *Acinetobacter baumannii*, and *Proteus mirabilis.*

## Data Availability

All data generated or analyzed during this study are included in this published article.

## Abbreviations

SSTIs: Skin and soft tissue infections
CSSTIs: Complicated skin and soft tissue infections
CPCSSTIs: Complicated posterior cervical skin and soft tissue infections
DM: Diabetes mellitus
CT: Computed tomography
WBC: White blood cell
CRP: C-reactive protein
HbA1c: Glycated hemoglobin (HbA1c)
SD: Standard deviation.
